# Extended D3 Mesenterectomy in Small Bowel Cancer Treatment: Feasibility and Importance for Staging. First Results of a Prospective Clinical Trial

**DOI:** 10.1002/wjs.70550

**Published:** 2026-09-02

**Authors:** Erik Kjæstad, Espen Thiis‐Evensen, Bjørn Edwin, Arne Olav Bakka, Anne Helene Lilleaas, Yngve Thorsen, Johannes Kurt Schultz

**Affiliations:** ^1^ Department of Gastrointestinal Surgery Akershus University Hospital University of Oslo Lørenskog Norway; ^2^ Institute of Clinical Medicine University of Oslo Oslo Norway; ^3^ Department of Gastroenterology ENETS Neuroendocrine Tumor Centre of Excellence Oslo University Hospital, Rikshospitalet Oslo Norway; ^4^ Interventional Centre Gastrointestinal and Pediatric Surgery Oslo University Hospital‐Rikshospitalet Oslo Norway

**Keywords:** D3 mesenterectomy, lymphadenectomy, neuroendocrine tumors, siNET, small bowel cancer, surgical treatment

## Abstract

**Background:**

There is no consensus on surgical techniques in small bowel cancer, particularly regarding the extent of lymph node dissection and strategic choices according to tumor localization and histological type. Clearance of central lymph node metastases can be challenging, especially when small intestinal neuroendocrine tumor (siNET) metastases give rise to fibrosis and desmoplasia around the cranial parts of the superior mesenteric vessels. The aim of this study was to assess the feasibility and staging implications of new techniques of central (D3) lymphadenectomy.

**Material and Methods:**

The operative techniques in this prospective small bowel cancer cohort entailed central (D3) removal of all mesenteric tissue anterior and posterior to the superior mesenteric vessels up to the root of the mesentery. Preoperative 3D mapping of the vascular anatomy was performed to facilitate personalized surgery. A new resection classification was developed, based on bowel and mesentery involvement.

**Results:**

Among the 86 patients included, D3 lymphadenectomy was performed in 74 (86%). Small bowel resection was done in 40 (54%), and ileocecal or ileocolic resection in 34 (46%). There were five (7%) reoperations (two (3%) anastomotic leaks) and no 30‐day mortality. At histopathology, 60 siNET (81%), 5 small bowel carcinomas, 3 metastases of other origin, 1 benign lesion, 3 lymphomas, and 2 gastrointestinal stromal tumors (GIST) were diagnosed. Lymph node count of 71 patients (one benign, two lymphomas not counted) showed mesenteric lymph node metastases in 62 (87%) (median lymph node yield 33). Metastases to the D3 volume were found in 31 (44%) (median D3 lymph node yield 13). In the siNET‐group, 28/60 (47%) had D3 metastases, suggested in standard preoperative investigations in 12 (43%). Of all positive siNET lymph nodes, 173/509 (34%) were localized in the D3 volume. Upper/middle ileal siNET were D3 positive in 19/29 (66%) versus in 9/31 (29%) of terminal ileal siNET. All patients had mesenterial R0 resections.

**Conclusions:**

Central (D3) small bowel mesenterectomy can be performed with radicality and safety. The high proportion of central metastases that would remain in situ after traditional, less extensive (D2) surgery underlines the potential importance of D3 mesenterectomy for accurate staging and locoregional disease control. Subgroup analyses suggested differences in mesenteric dissemination between terminal ileal and more proximal small intestinal tumors.

## Graphical abstract

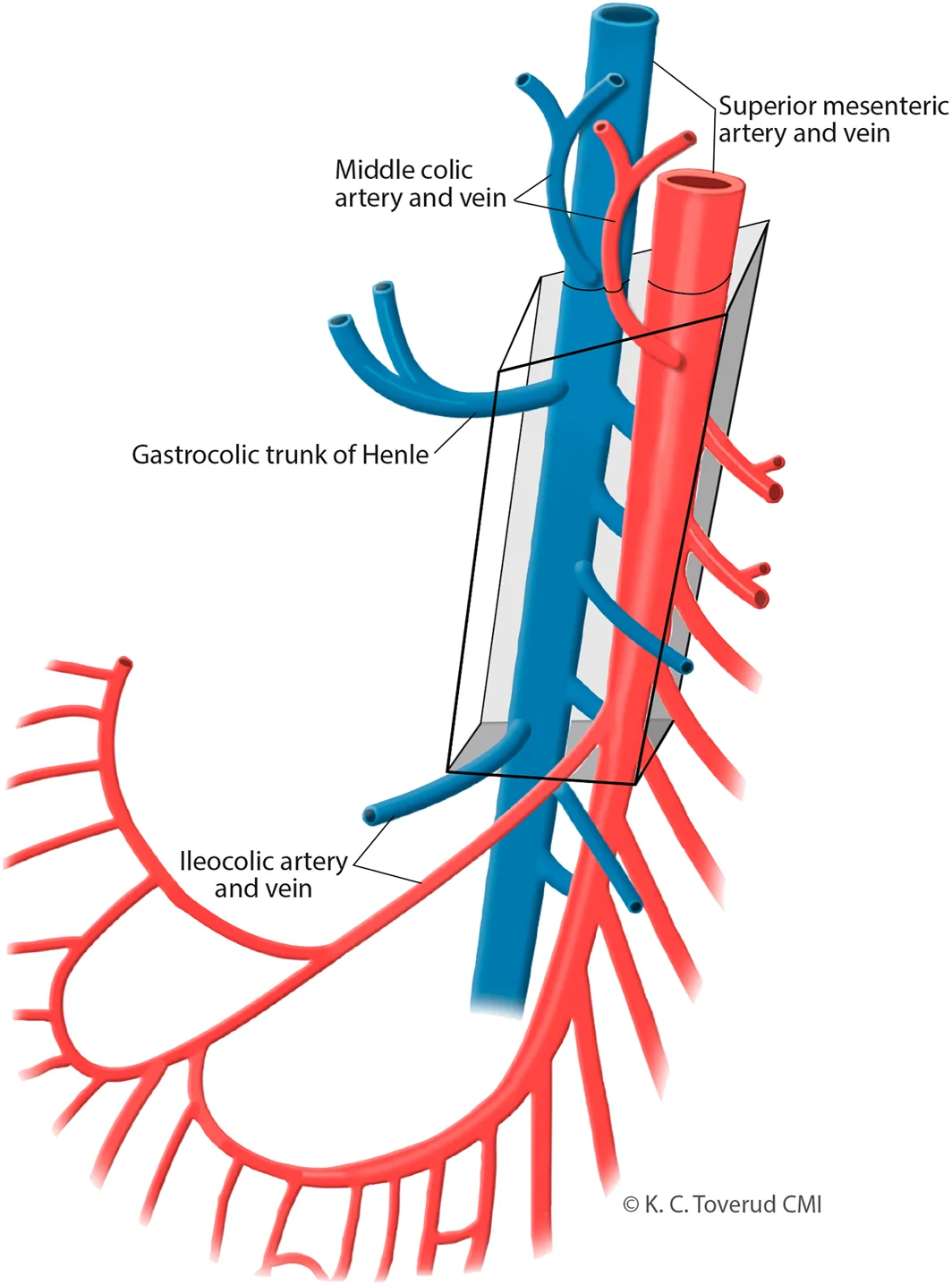

## 1 Introduction

Small bowel cancer (SBC) is rare, and accounts for less than 5% of all gastrointestinal malignancies [1, 2]. The incidence has more than doubled over the past few decades [3, 4]. SBC is a heterogeneous group of malignancies. Most frequent are small intestinal neuroendocrine tumors (siNET), followed by adenocarcinomas, lymphomas, and connective tissue tumors [5], all with a varied clinical course.

The recommended treatment for locoregional siNET and adenocarcinomas is surgical resection of tumor and lymph nodes [5, 6, 7, 8]. Lymph node retrieval has been linked to survival [9, 10, 11] but there is no consensus on the optimal extent of lymph node resection or which surgical techniques that should be used for the different tumor types [12, 13, 14, 15]. Small bowel lymphomas can give rise to intestinal obstruction, bowel perforation, and vascular occlusions requiring interventions, but the role of resection surgery for isolated small bowel lymphomas is not established [16]. About 20% of gastrointestinal stromal tumors (GIST) are located in the small bowel and the recommended treatment is bowel resection with limited lymph node dissection [17].

The extent of lymph node dissection must be balanced to the loss of bowel length [18] as more excessive lymph node resection usually entails division of more blood vessels, and loss of corresponding intestinal segments. In colon and gastric cancer surgery, mesenteric resection is classified as minimal (“near to bowel”, “D1”), intermediate (along vessel branches, “D2”), and central (around main vessel stems, “D3”) [19]. Lymph nodes can be removed with the lipolymphatic mesenterial tissue continuous with resected bowel parts in one piece (en‐bloc), or nodes can be picked one by one. En‐bloc resection is an established principle in gastrointestinal surgery to avoid spillage of cancer cells. Techniques to save blood vessels but gain more lymph nodes have emerged in colon cancer surgery [20] but are not developed to the same extent in siNET surgery. Especially the challenging treatment of central lymph node metastases, with fibrosis and desmoplasia surrounding the cranial parts of the superior mesenteric vessels, is debated [21, 22, 23, 24]. A classification of mesenteric siNET disease to evaluate resectability and assess risk versus benefit has been proposed [22, 25], but there still are unresolved issues of standardization, technical development, and consensus in siNET surgery [26].

The present study utilized experience from D3 colon cancer surgery to develop a new set of techniques for central (D3), en‐bloc, bowel‐length‐preserving lymphadenectomy in SBC, gaining lymph node clearance both anterior and posterior to the superior mesenteric vessels.

The aim of this prospective explorational (Phase IIS) study was to assess the feasibility and importance for staging of extended (D3) lymphadenectomy in SBC patients guided by preoperative 3D mapping of the vascular anatomy.

## 2 Material and Methods

### 2.1 Patients

Consecutive patients operated for SBC were offered prospective inclusion in the study. Inclusion criteria were patients with small bowel tumor(s) of probable or confirmed neoplastic nature above 18 years of age. Distal duodenal and duodenojejunal angle cancers were included; more proximal duodenal cancers were not. Surgery could be planned for curation, symptom relief, or debulking. Biopsy‐verified GIST or lymphoma patients were excluded. To evaluate the completeness of the inclusion, a board‐approved hospital database and the electronic journal system were searched for ICD‐10 codes for small bowel cancer and corresponding operative procedure codes during the study period.

### 2.2 Radiology and Vascular Anatomy Reconstruction

Preoperatively, all patients had their mesenterial arterial and venous vascular anatomy reconstructed in 3D by manual segmentation based on biphasic abdominal CT scans as previously described [20]. The standard preoperative imaging descriptions were evaluated for prediction of siNET D3 positivity.

### 2.3 Operative Technique

An inspection of the abdominal cavity for the extent of disease (cancer outside of resection field, synchronous intestinal tumors), and palpation of all small bowel and its mesentery was performed. In minimally invasive robotic or laparoscopic cases, a mini laparotomy was performed for exteriorization and palpation. The oncosurgical aim was an intact and continuous specimen with tumor and mesentery in one piece including unbroken anatomical tissue sheets. We defined the D3 volume to include all mesenteric tissue anterior and posterior to the superior mesenteric vessels' main stems limited by arterial and venous supply of the tumor‐bearing bowel segment, as described earlier in colon cancer surgery [20]. As illustrated in Figure 1, the anatomical landmarks were the superior mesenteric vein (SMV), the superior mesenteric artery (SMA), the middle colic artery (MCA), the first jejunal artery (FJA), and the ileocolic artery (ICA) origins, as well as the ileocolic vein (ICV) and the gastrocolic trunk of Henle (GTH) inflows. In cases of absent GTH [27], the middle colic vein (MCV) confluence with the SMV was used.

**FIGURE 1:**
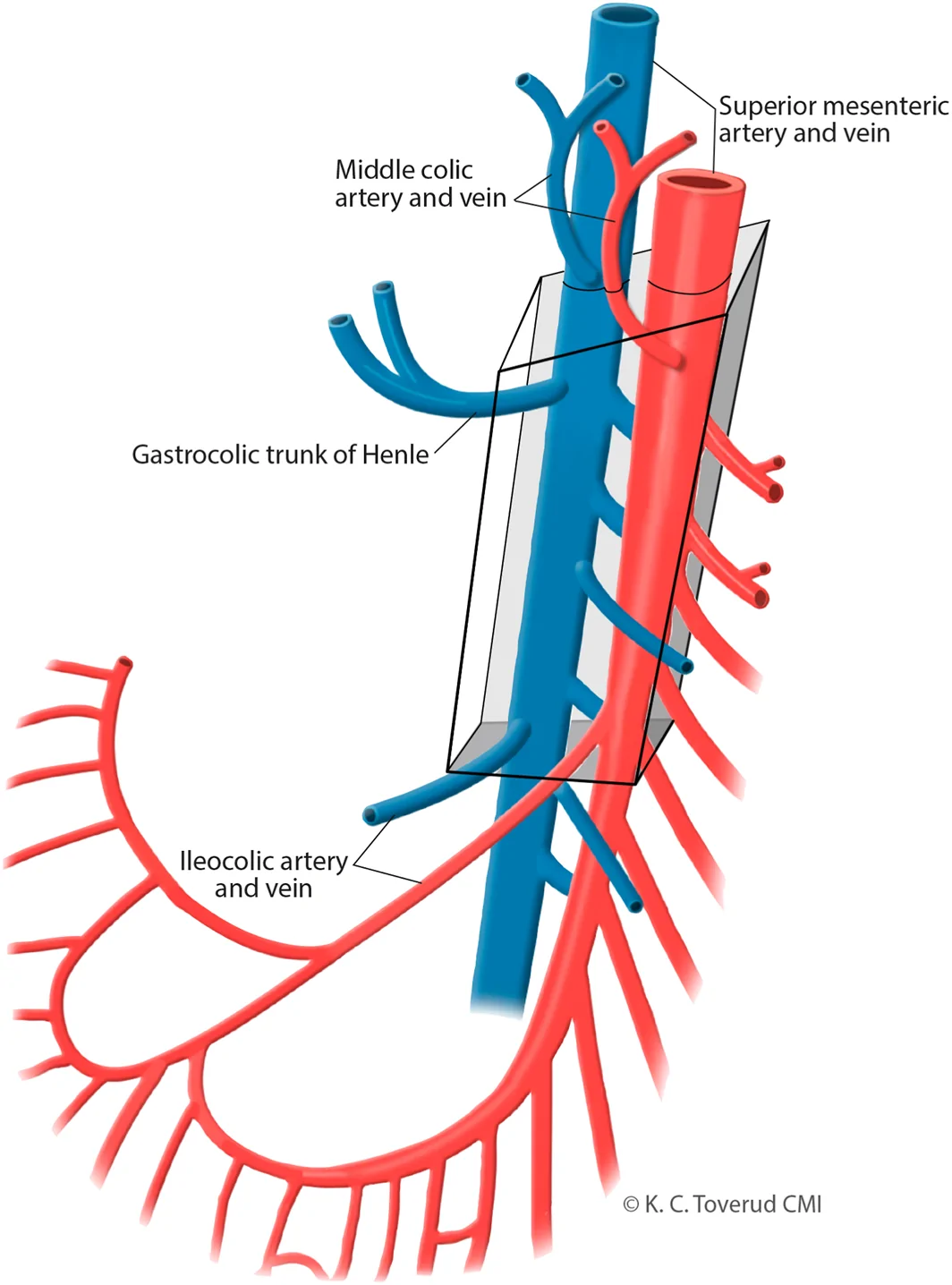
Central lipolymphatic tissue clearance both anterior and posterior to the superior mesenteric vessel stems. Definition of the D3 volume: Right‐side boundary: a line 1 cm to the right of the superior mesenteric vein (SMV). Left side border: a line along the left side of the superior mesenteric artery (SMA). Cranial border: a line 5 mm cranial to the line between the middle colic artery (MCA)^a^ origin from the SMA and the confluence of the gastrocolic trunk of Henle (GTH) with the SMV^b^. Caudal border: a line 1 cm caudal to the line between the ileocolic artery (ICA) origin from the SMA and the inflow of the ileocolic vein (ICV) into the SMV. ^a^For tumors at the duodenojejunal angle (DJA) of Treitz, the cranial border is defined to be 5 mm cranial to the first jejunal artery (FJA) if this origin is cranial to the MCA. ^b^In cases where there is no GTH, the middle colic vein (MCV) confluence with the SMV is used. The anterior border is the visceral peritoneum, and the posterior border is the retromesentric fascial plane.

A 3‐sector model of resection types, including two subgroups, was used, defined by the vessels involved in the disease: Type 1 (ICA) for tumors in the terminal ileum, type 2a (terminal SMA) for middle ileum tumors, type 2b (ileal artery) for upper ileum tumors, type 2c (jejunal artery) for jejunal tumors, and type 3 (first jejunal artery) for tumors at the duodenojejunal angle (DJA). The DJA segment was defined as the fourth part of duodenum and the first 10 cm of the jejunum. Type 3 operations were performed as intestinal derotation procedures to secure access to the first jejunal artery as modifications of known similar techniques [28]. Schematic examples of resection types are given in Figure 2. All fat and lymphatic tissue in the mesentery around the feeding vessels of the tumor segment was resected and included the whole width of the mesentery until the next jejunal or ileal blood vessels to each side of feeding vessels and mesenteric deposits. If, for example, ileal arteries number 2 and 3 were supplying the tumor segment, dissection was carried out along—but leaving intact—arteries number 1 and 4. We defined the level of the ICA origin as the border between ileal and jejunal mesentery: arteries that origin caudal to the ICA origin were considered ileal arteries, and arteries that origin cranial to the ICA origin as jejunal arteries. The standard anastomosis was hand‐sewn. Duodenojejunal resections were anastomosed with a circular stapling device. Mesenteric nodal masses, fibrosis, and desmoplasia were carefully dissected from the underlying blood vessels by exposing and dividing the vessel sheets so that the central vessels themselves could be spared. The D3 volume was separated and sent separately to histopathological analysis.

**FIGURE 2:**
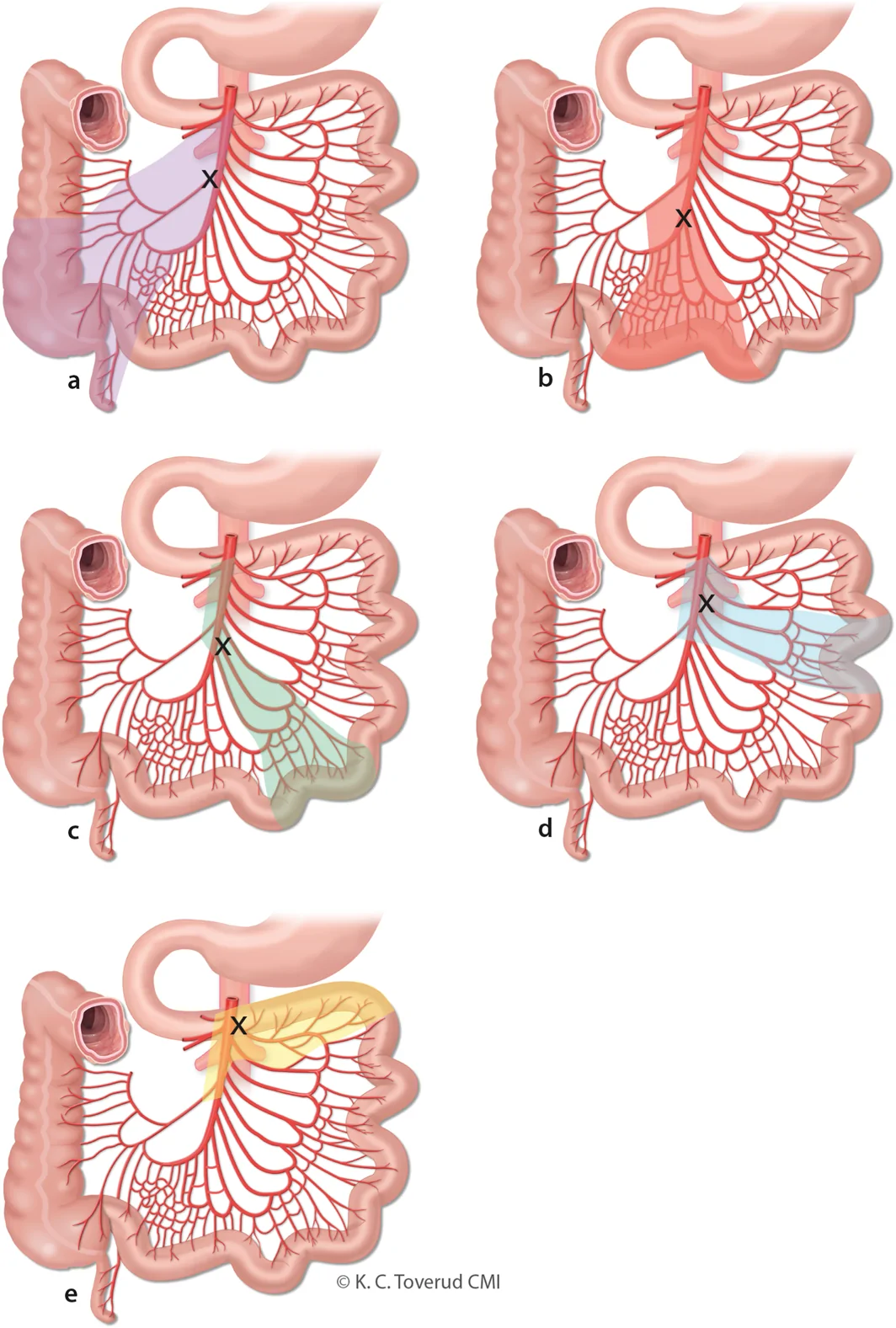
Examples of small bowel D3 resection types by feeding arteries. (a) Type 1: ileocolic artery (ICA) area (terminal ileum). (b) Type 2a: terminal superior mesenteric artery (SMA) (middle ileum). (c) Type 2b: ileal artery (upper ileum). (d) Type 2c: jejunal artery (jejunum). (e) Type 3: first jejunal artery (duodenojejunal flexure). Ileal arteries are caudal to the ICA origin, jejunal arteries cranial to the ICA origin. Duodenojejunal flexure = fourth part of duodenum and first 10 cm of jejunum. D3 volume is the same in all resections (see figure 2). In type 2b resections, the arcade connecting SMA and ICA is intact, in type 2a, the SMA is divided. Tumor feeding artery ligation is marked by an X. Mesenteric tissue around tumor‐feeding vessels is resected en bloc to the next adjacent jejunal or ileal branches, while uninvolved vessels are preserved to maintain bowel length.

### 2.4 Statistics

The data were analyzed regarding resection levels and types, tumor information including morphology, surgical outcomes including complication rates, and histopathological results, particularly the lymph node numbers retrieved. The IBM SPSS Statistics program, version 29.0 was used. Descriptive statistics were used in the calculations, and data were mainly described by medians and inter quartile ranges. Fisher's exact test and the chi square test were used to compare categorical variables, primarily to explore associations between D3 positivity and clinicopathological characteristics, acknowledging the limited statistical power of this exploratory cohort.

### 2.5 Ethics

The study was approved by the southeast Norwegian regional ethics committee (ID number 2016/1286). Written informed consent was collected from the patients.

## 3 Results

Altogether, 86 patients operated for suspected SBC from 14 November 2016 to 24 January 2023 were invited and consented to participate. D3 resection was feasible in 74 (86.0%) patients, all of whom had 3D vascular reconstructions based on preoperative abdominal CT scans. The patients not operated with D3 resection were prone to advanced cancer stages (*n* = 7) or intraoperative critical comorbidity (*n* = 1), or they had extensive adhesions (*n* = 1) or benign findings (*n* = 3). A study flow chart is presented in Figure 3. During the study period, three eligible patients were not approached for participation. The D3 volume could be delineated as described above (no cases of missing ileocolic arteries, all ileocolic veins entered the superior mesenteric vein). Minimally invasive access was used in 25/74 (33.8%) patients. Conversion to formal laparotomy was necessary in 4/25 (16.0%) patients due to bleeding (*n* = 1), adhesions (*n* = 1), or the need for extended palpation (*n* = 2). All patients were operated on by at least one of two project surgeons. All cases could be classified according to the new sector system. Resection type 1 was done in 31 (41.9%), type 2a in 22 (29.7%), type 2b in 10 (13.5%), type 2c in three (4.1%), and type 3 in five (6.8%) patients. In three (4.1%) patients, ligation of the ICA and the terminal SMA (type 1+ 2a) was performed. Due to primary tumor and mesenteric mass localizations, these were classified together with the other cases of terminal SMA involvement.

**FIGURE 3:**
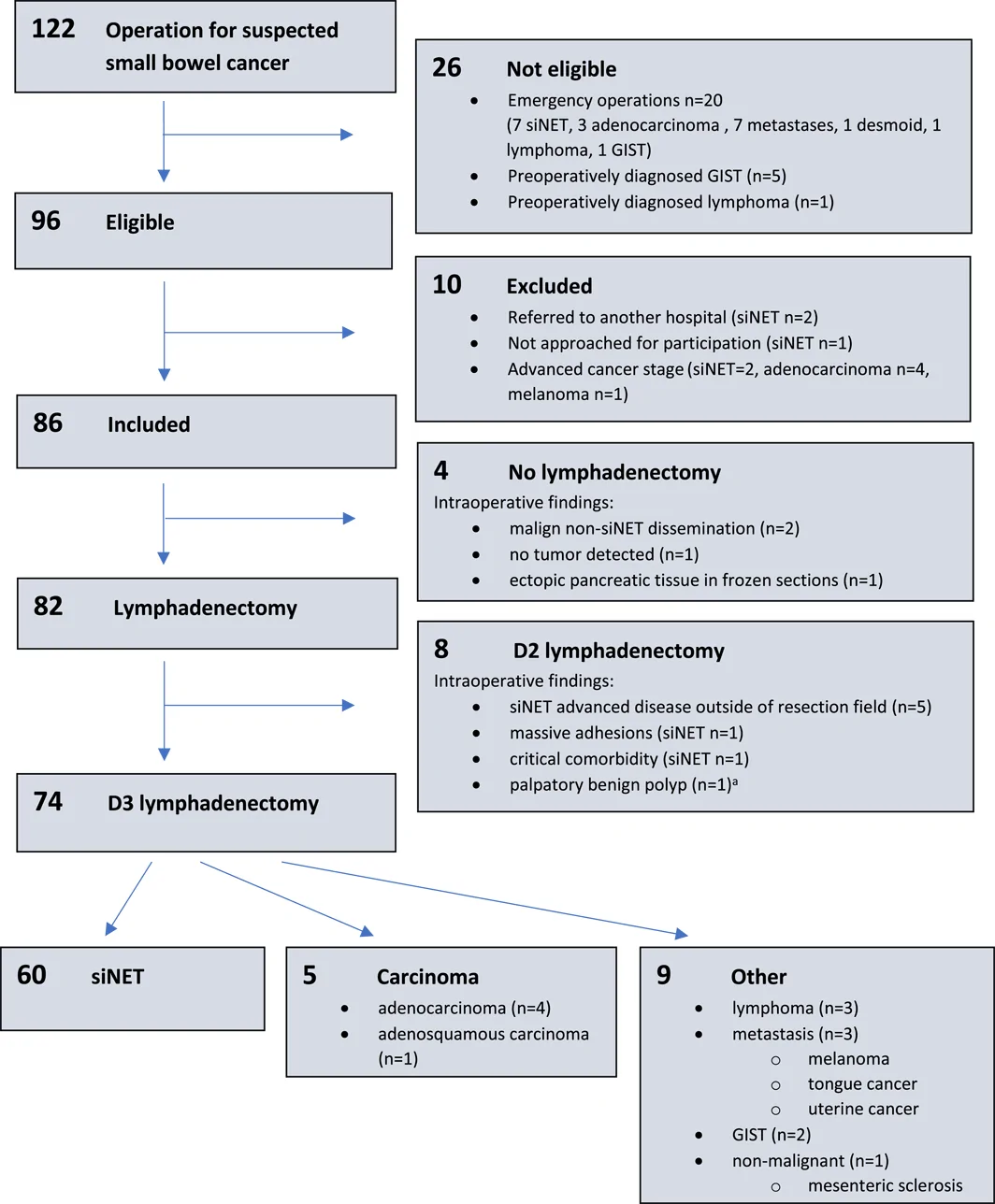
Study flow chart. GIST: gastrointestinal stromal tumor; siNET: small intestinal neuroendocrine tumors. ^a^Palpatory benign lesion, D3 surgery not justified.

Surgical results are presented in Table 1. Median operating time was 240 min (IQR 196–286), and median blood loss was 150 mL (IQR 100–275). Simultaneous resections were performed for siNET metastases involving the breast, liver, pancreas, uterus or uterine adnexa (one patient each), and for synchronous rectal and renal cancers (one patient each). Clavien‐Dindo classification of postoperative complications [29] grade 3a was registered for four (5.4%) and grade 3b for five (6.8%) (anastomotic leakage in two (2.7%)) patients. Grade 4 complications occurred in two (2.7%) patients with concomitant resection of pancreatic and rectal cancer (pancreatic leak, pelvic bleeding). The mortality at 30 days was zero. The median postoperative length of stay was 6 days (IQR 5–9).

**TABLE 1:** Baseline characteristics and surgical and histopathological results of the D3 cohort.

			Total D3 cohort (*n* = 74)	siNET (*n* = 60)	Carcinoma (*n* = 5)
Baseline characteristics					
Age: years—median (IQR)			60.5 (51.0–70.0)	59.0 (50.3–69.0)	65.0 (50.0–74.5)
Sex (female)			32 (43.2)	28 (46.7)	1 (20.0)
BMI: Kg/m^2^—median (IQR)			25.9 (23.9–30.2)	26.1 (24.3–30.8)	26.3 (24.2–35.7)
ASA class	1		8 (10.8)	6 (10.0)	0
	2		47 (63.5)	39 (65.0)	4 (80.0)
	3		19 (25.7)	15 (25.0)	1 (20.0)
	4		0	0	0
Previous abdominal surgery			47 (63.5)	39 (65.0)	3 (60.0)
Surgical data					
Operative time: min—median (IQR)			240 (196–286)	240 (200–303)	244 (198–279)
Blood loss: ml—median (IQR)			150 (100–275)	100 (50–200)	250 (150–650)
Clavien‐dindo classification	4	Organ dysfunction/intensive care treatment^c^	2 (2.7)	2 (3.3)	0
	3b	Anastomotic leakage	2 (2.7)	2 (3.3)	0
		Bleeding	2 (2.7)	1 (1.7)	1 (20.0)
		Ischemia	1 (1.4)	0	1 (20.0)
	3a	Abscess, ascites Myocardial infarction	3 (4.1)	2 (3.3)	0
			1 (1.4)	1 (1.7)	0
	2	Thromboembolism (femoral/lung) Superficial surgical site infection Anemia	2 (2.7)	2 (3.3)	0
			1 (1.4)	1 (1.7)	0
			1 (1.4)	1 (1.7)	0
Mortality 30 days			0	0	0
POLS: days—median (IQR)			6 (5–9)	6 (5–8)	6 (5–11)

		Total D3 cohort (*n* = 71)	siNET (*n* = 60)		Carcinoma (*n* = 5)	
Histopathological data						
Overall	Number of lymph nodes in total per patient—median (IQR)	33.0 (25.0–42.0)	34.0 (27.0–42.8)		25.0 (21.0–31.0)	
	Number of patients with positive lymph nodes	62 (87.3)	56 (93.3)		4 (80.0)	
	Number of positive lymph nodes in *N*+ cases—median (IQR)	6.5 (3.0–11.0)	7.0 (4.0–11‐0)		2.0 (1.3–2.0)	
	Lymph node ratio in *N*+ cases—median (IQR)	0.2 (0.1–0.4)	0.2 (0.1–0.4)		0.1 (0.1–0.1)	
D3 volume	Number of D3 lymph nodes in total per patient—median (IQR)	13.0 (9.0–19.0)	12.5 (9.0–18.0)		15.0 (8.5–19.5)	
	Proportion of positive lymph nodes located in D3 volume	179/574 (31.2)	173/509 (34.0)		1/7 (14.3)	
	Number of patients with positive D3 lymph nodes	31 (43.7)	28 (46.7)		1 (20.0)	
	Number of positive D3 lymph nodes in D3 *N*+ cases—median (IQR)	3.0 (1.0–8.0)	4.0 (2.0–9.5)		1	
	Lymph node ratio in D3 *N* + cases—median (IQR)	0.3 (0.2–0.8)	0.3 (0.2–0.8)		0.1	
Mesenteric R0 resections		74 (100.0)	60 (100.0)		5 (100.0)	
Differentiation			Grade 1	57 (95.0)	Well differentiated adenocarcinoma	2
					Medium differentiated adenocarcinoma	1
			Grade 2	3 (5.0)	Poorly differentiated adenocarcinoma	1
					Adenosquamous carcinoma	1
TNM classification		T0	1 (1.7)			
		T1	6 (10.0)			
		T2	11 (18.3)			
		T3	12 (20.0)		1 (20.0)	
		T4	30 (50.0)^b^		4 (80.0)	
		N0	4 (6.7)		1 (20.0)	
		N1	26 (43.3)		4 (80.0)	
		N2	30 (50.0)^a^			
		M0	42 (70.0)		4 (80.0)	
		M1	18 (30.0)		1 (20.0)	
Tumor diameter: mm—median (IQR)			17.0 (12.0–21.0)		40.0 (31.0–50.0)	

Abbreviations: ASA = American Society of Anesthesiologist's Physical Status Classification System, BMI = Body Mass Index, POLS = post operative length of stay, siNET = small intestinal neuroendocrine tumors.

^a^ Of siNET N2 mesenteries, 30 (100.0%) had mesenteric masses > 2 cm, 13 (43.3%) had ≥ 12 positive nodes.

^b^ Of siNET T4 tumors, 30 (100.0%) were ≤ 4 cm in size.

^c^ There was one grade 4 complication each due to concomitant pancreatic and rectal cancer surgery. Values are *n* (%) unless otherwise indicated. Lymph node ratio = number of positive nodes/total number of nodes. Histopathological data available in 71/74 patients.

siNET were found in 67/86 (77.9%) of included patients and primary small bowel carcinomas in five (5.8%). The remaining tumor types are shown in Figure 3. For three patients (lymphoma *n* = 2, non‐malignant *n* = 1), a quantitative lymph node analysis was not performed. Complete D3‐volume analyses exist for 71 patients (tumor distribution in Figure 4, intraoperative pictures in Figure 5, and lymph node analyses in Table 1). Mesenteric lymph node metastases were histopathologically verified in 62/71 (87.3%). Median total lymph node yield was 33 nodes (range 8–97). Median D3 lymph node yield was 13 (range 3–58). Metastases in the D3 volume occurred in 31/71 (43.7%) patients. All these also had D2 metastases. An intraoperative photo of a specimen with the corresponding central mesenterial resection is shown in Supporting Information S1: Figure 1. The retromesenteric access to the superior mesenteric vessels is illustrated in Supporting Information S1: Figure 2.

**FIGURE 4:**
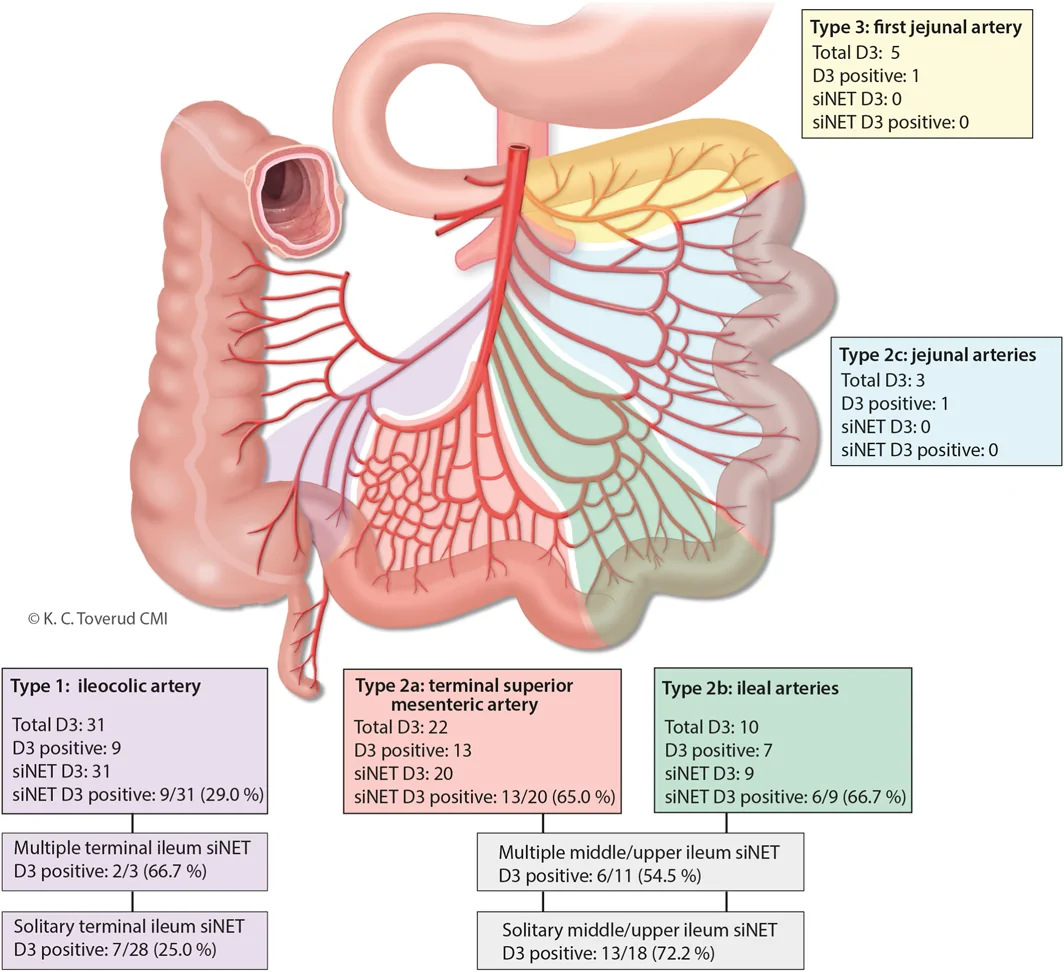
Distribution of D3 positives. Sectors of small bowel according to primary tumor feeding arteries and mesenteric mass location. (*n* = 71 with complete D3 data). Type 1: Ileocecal resection or right colectomy. Type 2: small bowel resection (2a: Middle ileum resection, 2b: Upper ileum resection, 2c: Jejunal resection). Type 3: Duodenojejunal resection. There is a tendency toward less extensive mesenteric dissemination in terminal ileal siNET, especially solitary tumors, which predominate in this area.

**FIGURE 5:**
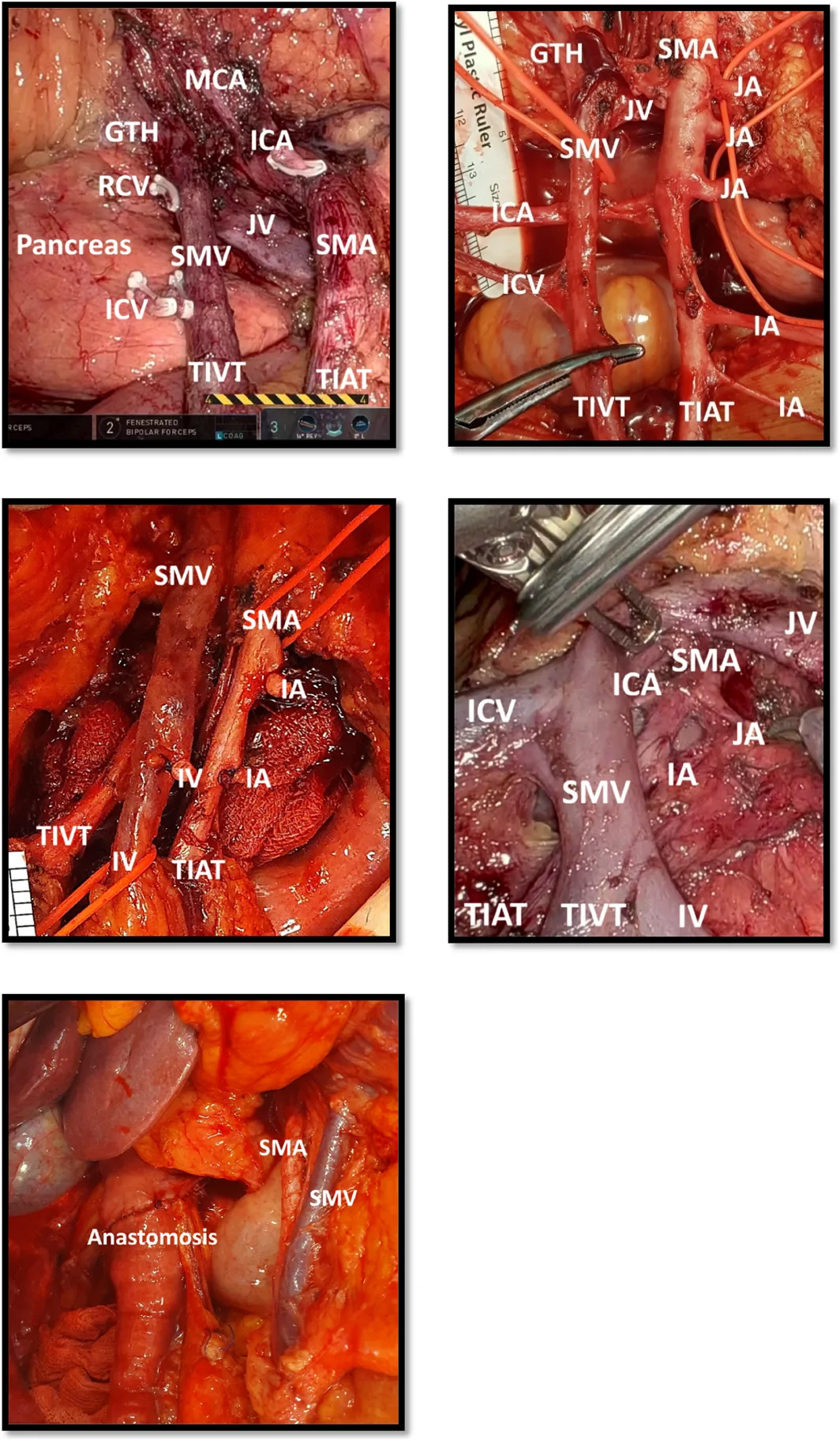
Intraoperative pictures. Examples of small bowel D3 resection types based on feeding arteries and draining veins. From top left: Type 1: ileocolic artery and vein. Type 2a: terminal superior mesenteric artery and vein. Type 2b: ileal arteries and vein. Type 2c: jejunal artery and vein (preparation, not ligated yet). Type 3: first jejunal artery (intestinal derotation). GTH: gastrocolic trunk of Henle; IA, IV: ileal artery, ‐vein; ICA, ICV: ileocolic artery, ‐vein; JA, JV: jejunal artery, ‐vein; MCA: middle colic artery; RCV: right colic vein; SMA, SMV: superior mesenteric artery, ‐vein; TIAT: terminal ileal arterial trunk; TIVT: terminal ileal venous trunk.

Complete lymph node data were retrieved for all siNET D3 patients, all with a lymph node yield of ≥ 8. The proportion of siNET patients with D3 positive lymph nodes was 28/60 (46.7%). Median number of siNET positive D3 lymph nodes was 4 (range 1–36), with a lymph node ratio of 0.3. Of all positive lymph nodes, 173/509 (34.0%) were localized in the D3 volume. The distribution of D3 positive siNET by localization is presented in Figure 4. Upper/middle ileal siNET were D3 positive in 19/29 (65.5%), and terminal ileum siNET in 9/31 (29.0%) (*p* < 0.007). There was no statistically significant correlation between D3 positivity and tumor size, grade, distant metastases, venous infiltration, or perineural infiltration. Solitary terminal ileum siNET were D3 positive in 7/28 (25.0%) versus 13/18 (72.2%) of solitary middle/upper ileum siNET (*p* < 0.005).

There were no siNET patients with unresected mesenteric disease (R0 resection in 60 (100.0%)). There were remaining distant metastases in 14 (23.3%) patients (nine cases of liver metastases, four of carcinomatosis, and one each of lymph node metastases in retroperitoneum or mediastinum). Of 28 siNET patients with D3 volume metastases, 23 (82.1%) had an M0 status. Of the remaining five, one had a mediastinal metastasis, one had retroperitoneal metastases, and three had liver metastases. Central mesenteric metastases were suggested in standard preoperative investigations in 12 (42.9%) of the histopathologically D3 positive siNET patients (Supporting Information S1: Table 1).

Of the adenocarcinoma patients, one in five had histopathologically positive D3 nodes.

## 4 Discussion

The most important finding in this study is that patients with small intestinal cancers, especially siNET patients, often have central (D3) mesenteric metastases. These can be removed in a safe and radical manner with surgical techniques adopted from colon and pancreatic cancer surgery, refined with preoperative reconstructions of the vascular anatomy.

The main assumption of the project is that lymphatic drainage converges along the great vessels, the mesenteric root representing a bottle neck [30]. Central lymphatic spread in this area has been considered difficult to resect, especially in cases of siNET metastases prone to mesenterial inflammation and fibrosis [12]. The experience from D3 colon cancer surgery is that preoperative planning based on 3D reconstruction of vascular anatomy facilitates safe and radical central dissection [31, 32]. Postoperative diarrhea seems to subside over time [32]. Whether small bowel D3 resection induces more diarrhea postoperatively than conventional resection will be addressed in a follow‐up of our patients. The present project's customized surgery—with resection types defined by tumor and mesenteric masses localizations—was done with acceptable complication rates and perioperative morbidity. The anastomotic leak frequency was low (3%); in elective colon cancer surgery, 3%–8% leakage rates are considered acceptable [33]. The procedure must, however, be regarded as time‐consuming, although the material included outliers with complex simultaneous resections. Minimally invasive SBC surgery, as used in 34% of the cases in this study, has previously been described [34, 35]. Central mesenterectomy could be more challenging in obese patients. The average body mass index (BMI) of 25.9 kg/m^2^ in the D3 surgery cohort equals the average BMI of adult Norwegians (25.8 kg/m^2^), a value significantly lower than in many other Western countries [36].

The high proportion of metastases in the D3 volume, higher than we observe in colon cancers, has previously not been described. As many as 44% of patients had lymph node metastases in the D3 volume, who comprised a median of 13 nodes. There were no skip metastases, that is, no D3 cases without D2 positivity, as have been reported by others [37]. When we investigated the siNET subgroup, the tendency was even clearer; D3 metastases were revealed in 47% (28/60) of patients. Of all positive mesenterial siNET nodes, one third were in the D3 volume. The D3 resection entailed removal of all known disease (R0 and M0 status) in most patients. As we found in more than half of the siNET D3 positive cases, metastatic nodes can be preoperatively unknown despite thorough work‐up including contrast‐enhanced CT‐/MRI‐scans and somatostatin receptor imaging like ^68^Ga‐DOTATOC‐PET‐CT [38, 39]. We found a tendency toward less extensive mesenteric dissemination in terminal ileal siNET, especially for solitary tumors, compared to more proximal ileal siNET.

An interpretation of the present results is that if patients were to be operated using the currently most used surgery—bowel resection and D2 lymph node resection—almost half of the patients would have had remaining metastatic nodes in their central mesentery *after* surgery. The clinical effects of this are yet to be clarified, and long‐term outcomes of the project may elucidate the question.

The benefit of removing mesenteric disease in locoregional siNET is well documented, for correct staging [40, 41], symptom prevention and control [18, 37, 42], and, probably, for improved survival [40, 43]. The possibility of recurrence in unresected mesenteric nodes has been demonstrated [44] and the retrieval of ≥ 8 lymph nodes has been correlated to both survival benefits [45] and correct staging [40]. Mesenteric siNET metastases may be tumor deposits or lymph node metastases which may be fused in a mesenteric mass making the lymph node count difficult [46]. Even though well differentiated siNET typically show a slow growth rate, expanding mesenteric lymph node masses may cause displacement of other organs and inflammatory response and fibrosis, which can compromise bowel and blood vessels [47, 48]. Chronic pain and impaired quality of life as well as dramatic and acute presentations from vascular disaster or bowel obstruction may follow from lack of locoregional disease control. Distant siNET metastases are most frequently found in the liver [49]. Resection of primary tumor and mesenteric disease has been recommended in cases where liver transplantation is considered [50]. The benefit of debulking resection in siNET, even in the presence of unresectable liver metastases, is well documented [12, 49, 51, 52, 53, 54, 55]. Re‐operative mesenterial surgery for patients *earlie*r treated with less extensive lymph node resection with residual or recurrent mesenterial disease seems reasonable [56, 57].

For adenocarcinoma of the small bowel, positive survival effects of lymph node dissection have been demonstrated [5, 10]. The natural history of adenocarcinoma differs from that of siNET, and their tendency to dissemination and shortened lifespans is also more pronounced than for adenocarcinomas of the colorectum. As for other cancer types, one of the most predictable prognostic markers for this disease is lymph node removal [5].

This study has several limitations. First, sample sizes restrict statistical power and generalizability of subgroup analyses. Second, the single‐center design in a specialized setting with extensive experience in mesenteric surgery and siNET management may not reflect outcomes in less specialized environments. Third, the cohort includes a spectrum of tumor entities. This mirrors real‐world practice, where surgery is often planned based on imaging rather than preoperative tissue samples. Finally, only short‐term outcomes are reported here; long‐term data on recurrence patterns, mesenteric failure, and survival are awaited after sufficient observation time to clarify the true oncological impact of D3 mesenterectomy in SBC treatment.

The strength of the present study is the continuous inclusion of patients operated at the same hospital, by the same team, with tissue samples analyzed at the same pathology department. The same set of surgical techniques and anatomy reconstruction methods were applied to all patients. A study of 60 siNET patients might seem like a small study, but large cohort studies are usually register studies. These often lack granularity, especially regarding surgical approaches, that we can report in our study. The ENETs Centre of Excellence for Neuroendocrine Tumors secured quality control regarding the indication for surgery on the siNET patients.

The patient population suffering from small bowel cancer is limited. Exactly how many patients who would benefit from central mesenterectomy are unknown. In Norway, with a population of 5.5 million, 245 new cases of SBC are reported yearly, but this includes duodenal cancers which count for more than half [58, 59]. About one‐third of patients having GIST, metastases, disseminated lymphoma, or inoperability—as well as emergency surgery cases—will not be candidates for extended lymph node surgery. siNET incidence has been reported around 1/100.000 in the US and Europe [60, 61]. Potential positive effects of gathering these patients for treatment in high‐volume centers have been discussed [12, 18, 26, 41].

## 5 Conclusion

This small bowel cancer D3 study indicates that central mesenterial metastases can be safely resected with preoperative vascular anatomy mapping and personalized surgical strategies. A large number of patients with small intestinal neuroendocrine tumors have central mesenterial metastases, not detected preoperatively, and not resected in traditional standard surgery. The prognostic significance of this awaits further studies, as does the apparent subgroups of small intestinal neuroendocrine tumors.

## Author Contributions

**Erik Kjæstad:** conceptualization, investigation, writing – original draft, methodology, validation, visualization, writing – review and editing, data curation, resources, project administration, software, formal analysis. **Espen Thiis‐Evensen:** conceptualization, investigation, writing – original draft, methodology, writing – review and editing, project administration, supervision, resources. **Bjørn Edwin:** supervision, writing – original draft, writing – review and editing, conceptualization. **Arne Olav Bakka:** conceptualization, investigation, writing – original draft, writing – review and editing, validation, supervision, resources, methodology. **Anne Helene Lilleaas:** investigation, writing – review and editing, software, formal analysis, validation, data curation. **Yngve Thorsen:** conceptualization, investigation, writing – review and editing, supervision, project administration, methodology. **Johannes Kurt Schultz:** writing – review and editing, visualization, supervision, validation, project administration, methodology, investigation, resources.

## Funding

The authors have nothing to report.

## Ethics Statement

Informed consent was obtained from all individual participants included in the study. The study was approved by the southeast Norwegian regional ethics committee (ID number 2016/1286).

## Conflicts of Interest

The authors declare no conflicts of interest.

## Supporting information


Supporting Information S1

## Data Availability

The data that support the findings of this study are available on request from the corresponding author. The data are not publicly available due to privacy or ethical restrictions.
