# Supplementary material for: Extended D3 Mesenterectomy in Small Bowel Cancer Treatment: Feasibility and Importance for Staging. First Results of a Prospective Clinical Trial
Source: World J Surg. 2026 Sep 2;50(10):2996–3007. doi: 10.1002/wjs.70550 (PMC13662501; doi:10.1002/wjs.70550)
Supplement: Supplementary file 1 [file WJS-50-2996-s001.docx]

**Extended D3 mesenterectomy in small bowel cancer treatment, feasibility and importance for staging: short-term results of a prospective clinical trial.**

**Supplementary figure 1**. **Specimen** example including lipolymphatic “tongue” anterior and posterior to the superior mesenteric vessels.

**Supplementary figure 2**. **Retromesenteric access** to the superior mesenteric vessels. SMV: superior mesenteric vein. JV: jejunal vein.

| **Preoperative lymph node involvement in siNET D3 positive patients (n=28)** | **Number of patients** |
| --- | --- |
| No mesenteric metastases on any investigation | 2 (7.1) |
| Metastases restricted to D1 or D2 on CT alone | 3 (10.7) |
| Metastases restricted to D1 or D2 on SRI alone | 0 |
| Metastases restricted to D1 or D2 on CT *and* SRI | 11 (39.3) |
| Metastases in D3 volume on CT alone | 2 (7.1) |
| Metastases in D3 volume on SRI alone | 2 (7.1) |
| Metastases in D3 volume on CT *and* SRI | 8 (28.6) |

**Supplementary table 1**. **Preoperative investigation** of small intestinal neuroendocrine tumor (siNET) histopathologically D3 positive patients. SRI: somatostatin receptor imaging. All patients had preoperative CT investigations, two did not have an SRI. Magnetic resonance imaging (MRI) was done in four patients with same results in this regard as the corresponding CT. When not explicit noted, D1 or D2 status was stated for descriptions of local, bowel-near and peripheral nodes at branches of the superior mesenteric artery (SMA) or vein (SMV), and D3 status for nodes described to be central, at the mesenterial root, at SMA or SMV main stems. Values are n (%).

**LEGEND**

**Supplementary figure 1**. **Specimen** example including lipolymphatic “tongue” anterior and posterior to the superior mesenteric vessels.

**Supplementary figure 2.** **Retromesenteric access** to the superior mesenteric vessels. SMV: superior mesenteric vein. JV: jejunal vein.

**Supplementary table 1**. **Preoperative investigation** of small intestinal neuroendocrine tumor (siNET) histopathologically D3 positive patients. SRI: somatostatin receptor imaging. All patients had preoperative CT investigations, and two did not have an SRI. Magnetic resonance imaging (MRI) was done in four patients with same results in this regard as the corresponding CT. When not explicit noted, D1 or D2 status was stated for descriptions of local, bowel-near and peripheral nodes at branches of the superior mesenteric artery (SMA) or vein (SMV), and D3 status for nodes described to be central, at the mesenterial root, at SMA or SMV main stems. Values are n (%).
